# Performance evaluation of antigen detection rapid diagnostic test (Ag-RDT) for COVID-19 diagnosis in a primary healthcare center during the Shanghai COVID-19 quarantine period

**DOI:** 10.1186/s12985-022-01871-6

**Published:** 2022-09-01

**Authors:** Lan Dong, Wen-Fang Li, Ying Jiang

**Affiliations:** 1 Department of Emergency Department, Shanghai Chang Zheng Hospital, Shanghai, People’s Republic of China; 2 Department of Neurosurgery, Shanghai Chang Zheng Hospital, Shanghai, People’s Republic of China; 3 Cerebrovascular Diseases Center, Department of Neurosurgery, Renji Hospital, Shanghai, People’s Republic of China

**Keywords:** COVID-19, SARS-CoV-2, Antigen rapid diagnostic test (Ag-RDT), Sensitivity, N gene, ORF1ab gene

## Abstract

**Background:**

Rapid and accurate detection of SARS-CoV-2 infection is the cornerstone of prompt patient care. However, the reliability of the antigen rapid diagnostic test (Ag-RDT) in the diagnosis of SARS-CoV-2 infection remains inconclusive.

**Methods:**

We conducted a field evaluation of Ag-RDT performance during the Shanghai Coronavirus disease 2019 (COVID-19) quarantine and screened 7225 individuals visiting our Emergency Department. 83 asymptomatic SARS-CoV-2 (+) individuals were enrolled in the current study. Simultaneously, Ag-RDT was performed to evaluate its testing performance.

**Results:**

For the Ag-RDT(−) cases, the average cycle threshold (Ct) values of the N gene were 27.26 ± 4.59, which were significantly higher than the Ct value (21.9 ± 4.73) of the Ag-RDT(+) individuals (*p* < 0.0001). The overall sensitivity of Ag-RDT versus that of RT-PCR was 43.37%. The Ag-RDT(+) individuals regarding the N gene’s Ct value were 16 cases in the < 20 range, 12 in 20–25, 5 in 25–30, and 3 in 30–35. The corresponding sensitivity was 84.21%, 52.17%, 21.74% and 16.67%, respectively. Meanwhile, sampling had a straight specificity of 100% regardless of the Ct value.

**Conclusions:**

The Ag-RDT were extremely sensitive in asymptomatic COVID-19 individuals with a Ct value < 20.

## Background

On 11 March 2020, the World Health Organization (WHO) declared the outbreak of Coronavirus disease 2019 (COVID-19) as a public health emergency of international concern [1, 2]. The number of infected cases and deaths due to COVID-19 is rising alarmingly ever since. As of 15th May 2022, over 518 million confirmed cases and over 6 million deaths have been reported globally [3]. Thus, detecting severe acute respiratory syndrome coronavirus 2 (SARS-CoV-2) in both symptomatic and asymptomatic COVID-19 individuals is paramount to blunt the community transmission of COVID-19.

Rapid and accurate detection of SARS-CoV-2 infection in patients, especially for those requiring emergency medical attention, is the cornerstone of prompt patient care and contact tracing. Nowadays, the nucleic acid amplification test (NAAT), also known as the Real-time Polymerase Chain Reaction (RT-PCR), is the diagnostic reference standard for SARS-CoV-2 infection in clinical microbiology laboratories [4]. However, healthcare workers are at risk of SARS-CoV-2 infection during aerosol-generating procedures, such as nasopharyngeal swabbing, intubation, bronchoscopy, and sputum induction [5]. Additionally, the RT-PCR is a time-consuming process and required specialized laboratory infrastructures and capacity. On the other hand, the antigen detection rapid diagnostic test (Ag-RDT) is a rapid viral diagnosis method and requires only a comparatively short testing period. Moreover, the Ag-RDT is also less laborious and does not require expensive medical devices and complicated training. Given these results, the Ag-RDT has emerged as a valuable alternative to RT-PCR for the diagnosis of SARS-CoV-2 infection. However, reporting of Ag-RDT performance remains limited. Among the handful of literature, the sensitivity bias associated with the viral load leads to high heterogeneity in the reported performance parameters of Ag-RDT [6–11], which strongly depend on the disease status and sample origins of tested individuals.

From April 1st to May 7th, 2022, Shanghai was under a strict quarantine due to the COVID-19 outbreak and the COVID-19 screening test was performed during patients’ visits to the hospital to prevent COVID-19 transmission inside hospitals. Since there was only a limited amount of field evaluation of Ag-RDT in asymptomatic cases, we collected the results of screening tests in the Emergency Department to determine whether Ag-RDT could accurately reflect the presence of infectious status in asymptomatic SARS-CoV-2–positive individuals compared to RT-PCR methodology.

## Methods

### Patients

**Fig. 1:**
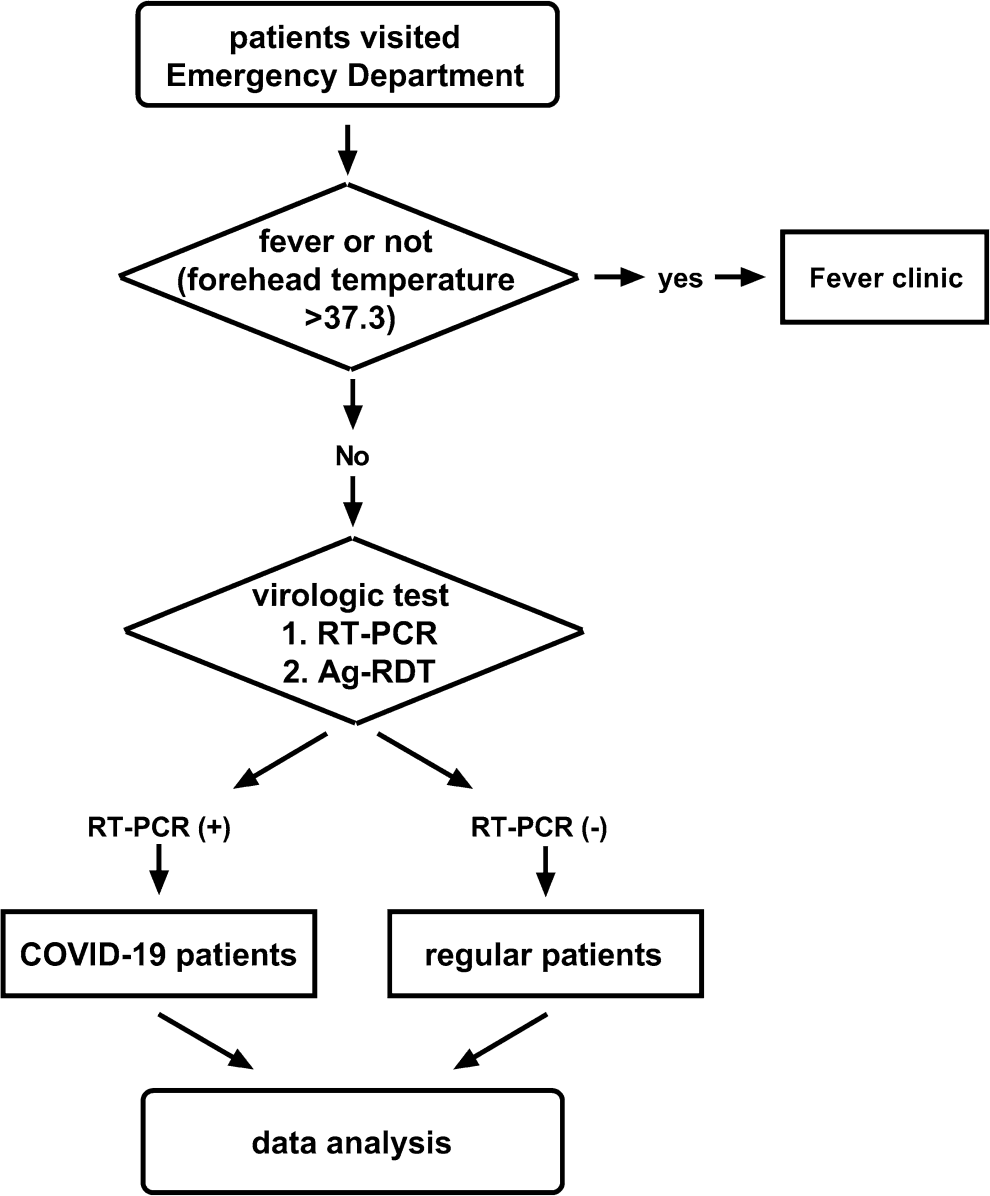
Flowchart of the approach used in the current study

This is a single-centered, retrospective observational study, conducted at our hospital from April 1st to May 7th, 2022, during which Shanghai (China) was under quarantine due to the COVID-19 outbreak. The flowchart of the study design process was available in Fig. 1. A total of 7225 consecutive individuals came to the Emergency Department of our hospital for medical care and received both RT-PCR and Ag-RDT during their visit. The asymptomatic SARS-CoV-2 (+) individuals were enrolled in the current study, who were defined as “individuals who test positive for SARS-CoV-2 using a virologic test (i.e., a NAAT or an antigen test) but who have no symptom that is consistent with COVID-19” [12]. The study was approved by the Insertional Research Ethics Committee and was carried out in accordance with The Code of Ethics of the World Medical Association (Declaration of Helsinki).

### Sample collection and SARS-CoV-2 testing

Using flocked swabs, trained nurses collected two respiratory specimens per patient (combined nasopharyngeal and throat swabs). One sample was used for Ag-RDT while the other was for RT-PCR.

Right after the sampling, the COVID-19 Ag-RDT assay (YHLO, Shenzhen, CN) targeting the N protein was performed immediately following the manufacturer’s instructions. After 15 min sample-Ag-RDT incubation time, the results were interpreted by the naked eye of trained nurses. In case of doubtful results, the nurse in chief interpreted the test results.

The RT-PCR samples were performed in accordance with the United States ECDC guidelines for oropharyngeal/nasopharyngeal testing. The samples were stored at 2–8 °C until testing, which was conducted within 6 h of sample collection. The COVID-19 Combo Kit (Zhijiang Biotechnology Co., Ltd., Shanghai, CN) targeting both SARS-CoV-2 open reading frame (ORF1ab) and nucleoprotein N (N genes) were used. The RT-PCR tests were performed by the Applied Biosystems™ 7500 Real-Time PCR Systems (Applied Biosystems, Pleasanton, CA, US) and the results were analyzed by the corresponding software. RT-PCR was considered positive if the cycle threshold (Ct) values of both the ORF1ab and N genes of SARS-CoV-2 were ≤ 35.

### Statistical analyses

Descriptive statistics were expressed as mean ± standard deviation (SD). Comparisons between two groups were performed using Student’s t-test for continuous variables. The GraphPad^®^ Prism 8.0 (GraphPad Prism Software Inc, California, CA, US) was used for statistical analyses. *p* values < 0.05 were considered statistically significant.

## Results

### Demographic and clinical information

Among the 7225 patients, a total of 83 patients were diagnosed with COVID-19 as their fresh respiratory swab samples were tested positive for SARS-CoV-2 by RT-PCR, which accounted for 1.15% of all emergency department visits. The mean and median age of these asymptomatic patients were 59.96 ± 21.09 and 64.5, respectively. Among them, the female gender accounted for 56.62% (47 cases) of the COVID-19 patients (Table 1). A total of 36 participants who tested positive for COVID-19 with the Ag-RDT were also positive with the SARS-CoV-2 RT-PCR, which represented 43.37% of the total participants. Meanwhile, none of the Emergency Department visitors (0 case) who tested negative with SARS-CoV-2 RT-PCR demonstrated positive results using the Ag-RDT.

**Table 1:** Demographic data of study participants

	Ag-RDT(−)	Ag-RDT(+)
*Gender*		
Male	21	15
Female	26	21
*Age*		
Mean ± SD	58.15 ± 19.85	62.03 ± 22.9
Median (IQR)	62 (43–72)	65 (46.5–79.5)

*SD* Standard deviation; *IQR* Interquartile range

### Laboratory findings

**Fig. 2:**
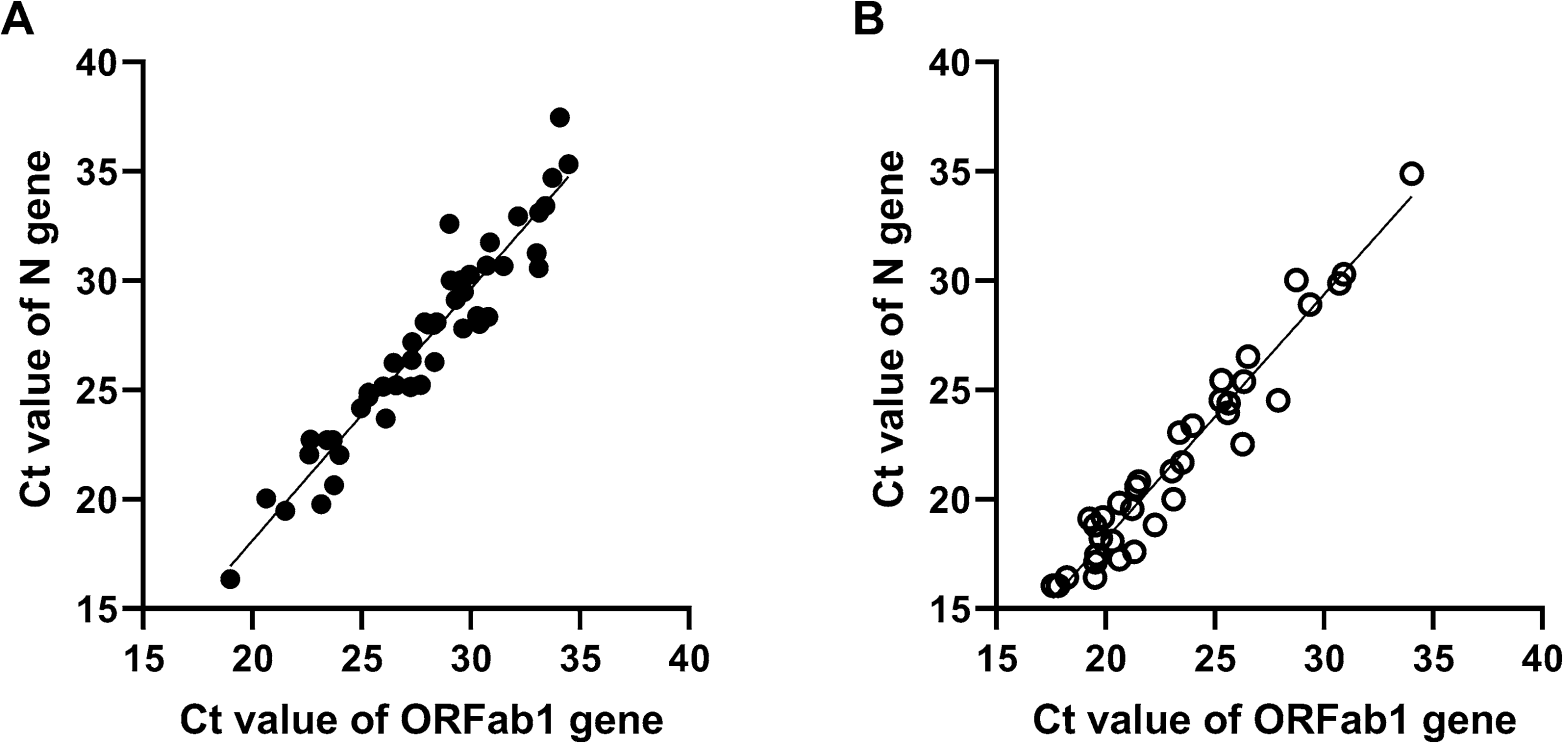
Pearson’s linear correlation between the ORF1ab and N genes. A positive linear correlation was observed between the cycle threshold (Ct) value of ORF1ab and N genes in both **A** Ag-RDT(−) group (slope = 1.15 + 0.05, R2 = 0.91) and **B** Ag-RDT(+) group (slope = 1.12 + 0.05, R2 = 0.94)

**Fig. 3:**
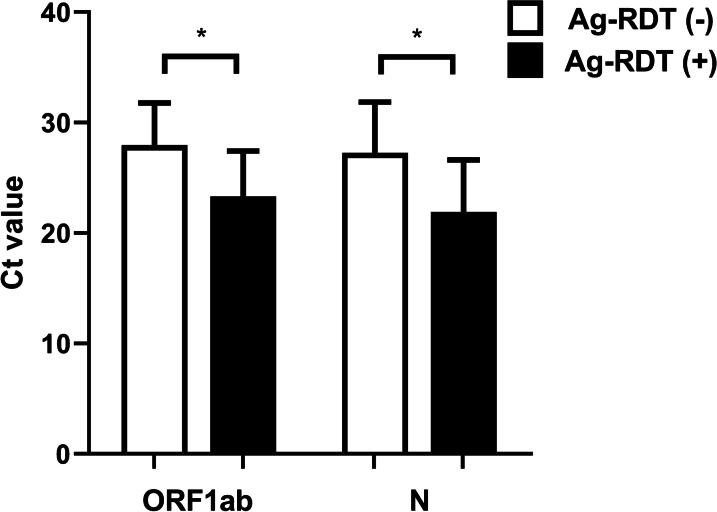
The comparison of ORF1ab and N genes expression between the Ag-RDT(−) and Ag-RDT(+) group. The Ag-RDT(−) group demonstrated significantly lower cycle threshold (Ct) values of ORF1ab and N genes than that of the Ag-RDT(+) group (both *p* < 0.0001)

The Ct value of ORF1ab and N genes correlated well with each other in both Ag-RDT(−) (slope = 1.15 ± 0.05, R^2^ = 0.91) (Fig. 2A) and Ag-RDT(+) groups (slope = 1.12 ± 0.05, R^2^ = 0.94) (Fig. 2B). For the Ag-RDT(−) cases, the average Ct values were 27.95 ± 3.82 for the ORF1ab gene (Table 2, Fig. 3), which were significantly higher than the Ct value (23.32 ± 4.1) of the Ag-RDT(+) group (*p* < 0.0001). Similarly, the average Ct values of the N gene were 27.26 ± 4.59 and 21.9 ± 4.73 in the Ag-RDT(−) and Ag-RDT(+) individuals, respectively (Table 3, Fig. 3). The difference between the two groups was also dramatic (*p* < 0.0001).

**Table 2:** The cycle threshold (Ct) value (± standard deviation) of the ORF1ab gene in respiratory specimens (combined nasopharyngeal and throat swabs) in different subgroups of SARS-CoV-2-RT-PCR confirmed cases

	Ag-RDT negative		Ag-RDT positive	
	Mean Ct value	n	Mean Ct value	n
*Ct value of ORF1ab gene*				
< 20	18.99	1	19.07 ± 0.85	10
20 to < 25	23.03 ± 1.26	10	22.03 ± 1.25	13
25 to < 30	27.85 ± 1.44	22	26.7 ± 1.47*	10
> 30	32.27 ± 1.47	14	32.47 ± 2.2	3
Overall	27.95 ± 3.82	47	23.32 ± 4.1***	36

The asterisk (* and ***) indicated significant differences between the Ag-RDT negative and positive groups, whose p value equaled 0.047 and < 0.0001, respectively

**Table 3:** Sensitivity of the antigen rapid diagnostic test (Ag-RDT) for SARS-CoV-2-RT-PCR confirmed cases

	Ag-RDT negative		Ag-RDT positive		Sensitivity (%)
	Mean Ct value	n	Mean Ct value	n	
*Ct value of N gene*					
< 20	18.54 ± 1.9	3	17.88 ± 1.26	16	84.21
20 to < 25	22.77 ± 1.55	11	22.57 ± 1.65	12	52.17
25 to < 30	27.23 ± 1.41	18	27.23 ± 2.06	5	21.74
> 30	32.32 ± 2.21	15	31.74 ± 2.74	3	16.67
Overall	27.26 ± 4.59	47	21.9 ± 4.73 ***	36	43.37

The cycle threshold (Ct) value (± standard deviation) of the N gene in respiratory specimens (combined nasopharyngeal and throat swabs) was presented in different subgroups of SARS-CoV-2-RT-PCR confirmed cases. The asterisk (***) indicated a significant difference between the two groups and the *p* value was < 0.0001

The overall specificity and sensitivity of Ag-RDT were 100% and 43.37% (Table 4), respectively. Furthermore, the Ag-RDT(+) individuals regarding the N gene’s Ct value was 16 cases in < 20 range, 12 in 20–25, 5 in 25–30, and 3 in 30–35 (Table 3). The corresponding sensitivity was 84.21%, 52.17%, 21.74% and 16.67%, respectively.

**Table 4:** The sensitivity and specificity results of the antigen detection rapid diagnostic test (Ag-RDT) among asymptomatic SARS−COV-2-infected individuals

	PCR(+)	PCR(−)
Ag-RDT(+)	36	0
Ag-RDT(−)	47	7142
	Sensitivity = 43.37%	Specificity = 100%

## Discussion

In the current research, we studied the sensitivity and specificity of Ag-RDT in asymptomatic COVID-19 cases in a field-based scenario during the Shanghai COVID-19 quarantine period. We found that the Ag-RDT showed a satisfied specificity (100%). Although the sensitivity was relatively low for the entire samples (43.37%), the Ag-RDT demonstrated a good sensitivity for the subset of individuals with an RT-PCR Ct value of the N gene in the < 20 range (84.21%). This result indicated that Ag-RDT could be a reliable alternative to RT-PCR for the rapid detection of the individual with a higher risk of infectivity in mass screening of asymptomatic populations.

Diagnostic testing for SARS-CoV-2 is a critical constituent of the COVID-19 prevention and control strategy. According to the WHO interim guidance, the RT-PCR has the best sensitivity and specificity and is recommended as the reference standard for SARS-CoV-2 diagnosis [13]. However, the RT-PCR is a time-consuming process and required specialized laboratory infrastructures and capacity. Thus, in the last two years, commercialized Ag-RDTs were introduced to the market, aiming to offer an opportunity to increase the availability and speed of testing in appropriate scenarios. According to WHO, eligible Ag-RDT for SARS-COV-2 needs to meet the minimum performance requirements of ≥ 80% sensitivity and ≥ 97% specificity [14]. Similarly, the European Center for Disease Prevention and Control also suggests the use of Ag-RDT with performance closer to RT-PCR, i.e., ≥ 90% sensitivity and ≥ 97% specificity [15]. However, while the overall specificity met the WHO suggested standard (99.6%, 95% CI 99.0–99.8%) [16], several commercialized Ag-RDTs demonstrated a lack of sensitivity. According to a recent Cochrane study enrolling eight studies, the average sensitivity of the commercialized Ag-RDT corresponds to only 56.2% (95% CI 29.5–79.8%) for the general population [16]. Specifically, for symptomatic SARS-COV-2-infected individuals, the average sensitivity was 72.0% (95% CI 63.7–79.0%) [17], which was higher than that of the asymptomatic individuals (58.1%, 95% CI 40.2–74.1%). Baro et al. [18] conducted a head-to-head comparison of five Ag-RDTs for SARS-CoV-2 asymptomatic infection. They found that all these Ag-RDTs acquired high specificity (all > 89%). However, the overall sensitivity of Abbott, Siemens, Roche, and Lepu kits were only 38.61%, 51.49%, 43.56%, 45.54%, and 28.71%, respectively. Among those who had lower Ct values (< 30), the corresponding sensitivity increased to 66.67%, 86.67%, 83.33%, 83.33%, and 70%, respectively, which, however, remained below the minimum performance requirements of WHO and European Center for Disease Prevention and Control. Within our panel of clinical samples, the Ag-RDT used in the current study was proved to be highly specific and the sensitivity parament was overall in line with the previous report.

Our study has several limitations. First, it is expected that the types of SARS-CoV-2 variants are emerging. Previous studies had already demonstrated the variable performance of Ag-RDTs against different SARS-CoV-2 variants [19, 20]. Since SARS-CoV-2 culture and sequencing were not performed in the current study, the exact sensitivity of the current Ag-RDTs remained inconclusive for different SARS-CoV-2 variants. According to the literature, the Omicron was the major viral strain during the study period [21, 22]. Thus, we believe that the results of the current study mainly reflected the performance of Ag-RDT targeting the Omicron. Secondly, this field evaluation was performed from April to May. Although Shanghai had a low circulation of other frequent respiratory viruses, this issue had not been fully excluded, which could affect the specificity of Ag-RDTs. Thirdly, it is worth mentioning that all the respiratory samples enrolled in the current study were collected by trained nurses. Since operating personnel has been previously suggested to affect the testing performance of Ag-RDT significantly [23], we expected to achieve a lower sensitivity of the Ag-RDT during the screening of the general population. Additionally, the moderate sample size is another limitation for accurately evaluating the test performance of Ag-RDT.

## Conclusions

In conclusion, we demonstrated that for asymptomatic SARS-CoV-2 infected patients, the Ag-RDT demonstrated a good sensitivity for individuals with N gene’s Ct value < 20. Although the Ag-RDT is less sensitive than RT-PCR in asymptomatic populations, careful selection of cohorts for testing can mitigate this limitation.

## Data Availability

The datasets generated and/or analyzed during the current study are not publicly available but are available from the corresponding author on reasonable request.

## Abbreviations

WHO: World Health Organization
COVID-19: Coronavirus disease 2019
NAAT: Nucleic acid amplification test
RT-PCR: Real-time polymerase chain reaction
Ag-RDT: Antigen detection rapid diagnostic test
ORF1ab: The open reading frame
N genes: Nucleoprotein N

## References

[1] COVID-19 Public Health Emergency of International Concern (PHEIC). https://www.who.int/publications/m/item/covid-19-public-health-emergency-of-international-concern-(pheic)-global-research-and-innovation-forum.

[2] WHO Director-General’s opening remarks at the media briefing on COVID-19 - 20 March 2020. https://www.who.int/director-general/speeches/detail/who-director-general-s-opening-remarks-at-the-media-briefing-on-covid-19---20-march-2020.

[3] Weekly epidemiological update on COVID-19 - 18 May 2022. https://www.who.int/publications/m/item/weekly-epidemiological-update-on-covid-19---18-may-2022.

[4] Overview of Testing for SARS-CoV-2, the virus that causes COVID-19. https://www.cdc.gov/coronavirus/2019-ncov/hcp/testing-overview.html.

[5] Hamilton GS (2021). Aerosol-generating procedures in the COVID era. Respirology.

[6] Porte L, Legarraga P, Vollrath V, Aguilera X, Munita JM, Araos R, Pizarro G, Vial P, Iruretagoyena M, Dittrich S, Weitzel T (2020). Evaluation of a novel antigen-based rapid detection test for the diagnosis of SARS-CoV-2 in respiratory samples. Int J Infect Dis.

[7] Lambert-Niclot S, Cuffel A, Le Pape S, Vauloup-Fellous C, Morand-Joubert L, Roque-Afonso AM, Le Goff J, Delaugerre C (2020). Evaluation of a rapid diagnostic assay for detection of SARS-CoV-2 antigen in nasopharyngeal swabs. J Clin Microbiol.

[8] Lindner AK, Nikolai O, Kausch F, Wintel M, Hommes F, Gertler M, Krüger LJ, Gaeddert M, Tobian F, Lainati F, et al: Head-to-head comparison of SARS-CoV-2 antigen-detecting rapid test with self-collected nasal swab versus professional-collected nasopharyngeal swab. Eur Respir J. 2021;57:2003961. 10.1183/13993003.03961-2020 PMC7736752 33303544

[9] Albert E, Torres I, Bueno F, Huntley D, Molla E, Fernández-Fuentes M, Martínez M, Poujois S, Forqué L, Valdivia A (2021). Field evaluation of a rapid antigen test (Panbio™ COVID-19 Ag Rapid Test Device) for COVID-19 diagnosis in primary healthcare centres. Clin Microbiol Infect.

[10] Wölfl-Duchek M, Bergmann F, Jorda A, Weber M, Müller M, Seitz T, Zoufaly A, Strassl R, Zeitlinger M, Herkner H (2022). Sensitivity and specificity of SARS-CoV-2 rapid antigen detection tests using oral, anterior nasal, and nasopharyngeal swabs: a diagnostic accuracy study. Microbiol Spectr.

[11] Chaimayo C, Kaewnaphan B, Tanlieng N, Athipanyasilp N, Sirijatuphat R, Chayakulkeeree M, Angkasekwinai N, Sutthent R, Puangpunngam N, Tharmviboonsri T (2020). Rapid SARS-CoV-2 antigen detection assay in comparison with real-time RT-PCR assay for laboratory diagnosis of COVID-19 in Thailand. Virol J.

[12] Clinical Spectrum of SARS-CoV-2 Infection. https://files.covid19treatmentguidelines.nih.gov/guidelines/section/section_43.pdf.

[13] Public health surveillance for COVID-19: interim guidance. https://www.who.int/publications/i/item/WHO-2019-nCoV-SurveillanceGuidance-2022.2.

[14] World Health Organization: Antigen-detection in the diagnosis of SARS-CoV-2 infection. 2021.

[15] Options for the use of rapid antigen tests for COVID-19 in the EU/EEA - first update. https://www.ecdc.europa.eu/en/publications-data/options-use-rapid-antigen-tests-covid-19-eueea-first-update.

[16] Dinnes J, Deeks JJ, Adriano A, Berhane S, Davenport C, Dittrich S, Emperador D, Takwoingi Y, Cunningham J, Beese S (2020). Rapid, point-of-care antigen and molecular-based tests for diagnosis of SARS-CoV-2 infection. Cochrane Database Syst Rev.

[17] Dinnes J, Deeks JJ, Berhane S, Taylor M, Adriano A, Davenport C, Dittrich S, Emperador D, Takwoingi Y, Cunningham J (2021). Rapid, point-of-care antigen and molecular-based tests for diagnosis of SARS-CoV-2 infection. Cochrane Database Syst Rev.

[18] Baro B, Rodo P, Ouchi D, Bordoy AE, Saya Amaro EN, Salsench SV, Molinos S, Alemany A, Ubals M, Corbacho-Monné M (2021). Performance characteristics of five antigen-detecting rapid diagnostic test (Ag-RDT) for SARS-CoV-2 asymptomatic infection: a head-to-head benchmark comparison. J Infect.

[19] Bourassa L, Perchetti GA, Phung Q, Lin MJ, Mills MG, Roychoudhury P, Harmon KG, Reed JC, Greninger AL (2021). A SARS-CoV-2 nucleocapsid variant that affects antigen test performance. J Clin Virol.

[20] Gandolfo C, Morecchiato F, Pistello M, Rossolini GM, Cusi MG (2021). Detection of SARS-CoV-2 N protein allelic variants by rapid high-throughput CLEIA antigen assay. J Clin Virol.

[21] Chen X, Yan X, Sun K, Zheng N, Sun R, Zhou J, Deng X, Zhuang T, Cai J, Zhang J (2022). Estimation of disease burden and clinical severity of COVID-19 caused by Omicron BA.2 in Shanghai, February–June 2022. MedRxiv.

[22] Chen Z, Deng X, Fang L, Sun K, Wu Y, Che T, Zou J, Cai J, Liu H, Wang Y (2022). Epidemiological characteristics and transmission dynamics of the outbreak caused by the SARS-CoV-2 Omicron variant in Shanghai, China: a descriptive study. MedRxiv.

[23] Preliminary report from the Joint PHE Porton Down & University of Oxford SARS-CoV-2 test development and validation cell: rapid evaluation of lateral flow viral antigen detection devices (LFDs) for mass community testing. https://www.ox.ac.uk/sites/files/oxford/media_wysiwyg/UK%20evaluation_PHE%20Porton%20Down%20%20University%20of%20Oxford_final.pdf.

